# Ichthyofauna of Ceará-Mirim River basin, Rio Grande do Norte State, northeastern Brazil

**DOI:** 10.3897/zookeys.715.13865

**Published:** 2017-11-13

**Authors:** Nathalia Kaluana Rodrigues da Costa, Roney Emanuel Costa de Paiva, Márcio Joaquim da Silva, Telton Pedro Anselmo Ramos, Sergio Maia Queiroz Lima

**Affiliations:** 1 Universidade Federal do Rio Grande do Norte, Centro de Biociências, Departamento de Botânica e Zoologia, Laboratório de Ictiologia Sistemática e Evolutiva, Campus Central, Lagoa Nova, CEP 59078-900, Natal, RN, Brazil; 2 Programa de Pós-Graduação em Sistemática e Evolução

**Keywords:** Mid-Northeastern Caatinga Ecoregion, coastal basin, fishes of Caatinga and Atlantic Forest, estuarine ichthyofauna, inventory

## Abstract

Ichthyological studies in coastal basins of the Mid-Northeastern Caatinga ecoregion were first conducted in the early 20^th^ century, including collections from the Ceará-Mirim River basin, in northeastern Brazil. Besides a few systematics and ecological studies, the knowledge on fishes from this watershed is still considered partial and restricted to the freshwater portion. Thus, the objective of this paper was to conduct a comprehensive ichthyological survey of the entire Ceará-Mirim River basin, from the headwaters to the estuarine area. Fish surveys were conducted from 2011 to 2016 using varied fishing gear, resulting in the record of 63 native species (24 freshwater, 15 estuarine, and 24 marine species) and two introduced species. Four species are putatively endemic to the ecoregion, and 48 consist of new records for the basin. According to the Brazilian’s threatened fish list, three species are currently classified as ‘vulnerable’ (*Megalops atlanticus*, *Hippocampus reidi* and *Mycteroperca bonaci*), four as ‘near threatened’ (*Kryptolebias hermaphroditus*, *Dormitator maculatus, Lutjanus sygnagris* and *L. jocu*) and three as ‘data deficient’ (*Cheirodon jaguaribensis*, *Mugil curema* and *Sphoeroides testudineus*). The Ceará-Mirim River basin does not have any protected areas and has been suffering multiple anthropogenic impacts, however the "Centro Tecnológico de Aquicultura" (Aquaculture Technological Center) of the Universidade Federal do Rio Grande do Norte (CTA/UFRN) at the lower portion of the basin may help in the conservation of the estuarine and estuarine fish species.

## Introduction

The Mid-Northeastern Caatinga freshwater ecoregion (MNCE) located in the extreme northeast Brazil comprises the drainages between the largest perennial rivers of the region, the São Francisco and Parnaíba (Albert et al. 2011, Rosa et al. 2003). When compared to adjacent ecoregions, its hydrographical network is simpler and composed of small to medium size basins. In addition, most of its rivers are intermittent due to the predominance of the semi-arid climate (Rosa et al. 2003). Their margins usually present xeric shrublands and thorny forests of the Caatinga vegetation, except for the humid highland enclaves (Rosa and Groth 2004), and for a narrow strip of land running along the eastern coast of Brazil that harbors fragmented remnants of Atlantic Forest. This strip extends from the State of Rio Grande do Norte to Alagoas in the MNCE (Rosa 2004).

One of the basins draining into the eastern coast of the MNCE is the Ceará-Mirim River basin, in the Rio Grande do Norte State, and presents an intermittent hydrological regimen in the upper and medium portions, while the lower stretch, located in the Atlantic Forest area, is perennial. This particular basin is of historic importance due to the "Stanford Expedition" specimen collection conducted in 1911. This expedition, led by the naturalist Edwin Chapin Starks, visited locations in northern and northeastern Brazil, and cataloged 11 fish species in the Ceará-Mirim River basin, including the original description of the armored catfish *Hypostomus pusarum* (Starks, 1913). Samples from this basin were also taken in 1933 by the "Departamento Nacional de Obras Contra Secas – DNOCS", in an effort by the "Comissão Técnica de Piscicultura do Nordeste do Brasil", institution managed by Rodolpho von Ihering, to study the region’s ichthyofauna (Canan 2011).

Rosa (2004) recorded 11 freshwater fish species at the Ceará-Mirim River basin, without providing a list, and this same number were presented by Nascimento et al. (2014) in an ichthyofauna inventory of the basins of the Rio Grande do Norte State, also based on secondary data. In addition, studies on trophic ecology of freshwater fishes (Andrade et al. 2000, Gurgel et al. 2005, Dias and Fialho 2009) were also conducted in the basin. Recently, a new species was described (*Serrapinnus potiguar*, Jerep and Malabarba 2014), and the record of a self-fertilizing mangrove killifish (*Kryptolebias hermaphroditus*) was reported (Lira et al. 2015). Such occurrences corroborate the need for a broad ichthyofaunal inventory at this particular basin.

The lower portion of this basin is included within the northern limits of the Atlantic Forest domain and presents a mangrove forest area of approximately 3.15 km² (0.12% of the basin) (Maia et al. 2006) that might serve as feeding, breeding and refuge grounds for both marine and estuarine fish (Osório et al. 2011). This mangrove forest also comprises the "Centro Tecnológico de Aquicultura – CTA" (Aquaculture Technological Center) of the Universidade Federal do Rio Grande do Norte – UFRN, an area of approximately 7.7 km^2^ that was previously used for shrimp farming. This area was incorporated to the UFRN facilities in 2007, to develop research, teaching and training courses on Biological Sciences. Although part of the lower portion of the basin is somewhat protected by the CTA/UFRN, the Ceará-Mirim River basin suffers the impact of anthropogenic activities. Among such activities are the intake of domestic sewage, fertilizers and agrochemicals, as well as siltation and the deforestation of riparian forests or mangroves that compromise, especially, the aquatic biota (Soares et al. 2010).

Considering that knowledge on the ichthyofauna of a basin is paramount to monitoring anthropic impacts, as well as to encourage the development of further fish studies and other academic activities, the objective of this paper was to inventory the ichthyofauna of the Ceará-Mirim River basin. One important goal of this list is to provide data on the status of commercially significant and introduced species (Leão et al. 2011, Nóbrega et al. 2015) relevant to management and conservation actions, and compare this data with previous studies on the basin (Starks 1913, Nascimento et al. 2014).

## Material and methods

### Study area

**Figure 1.**
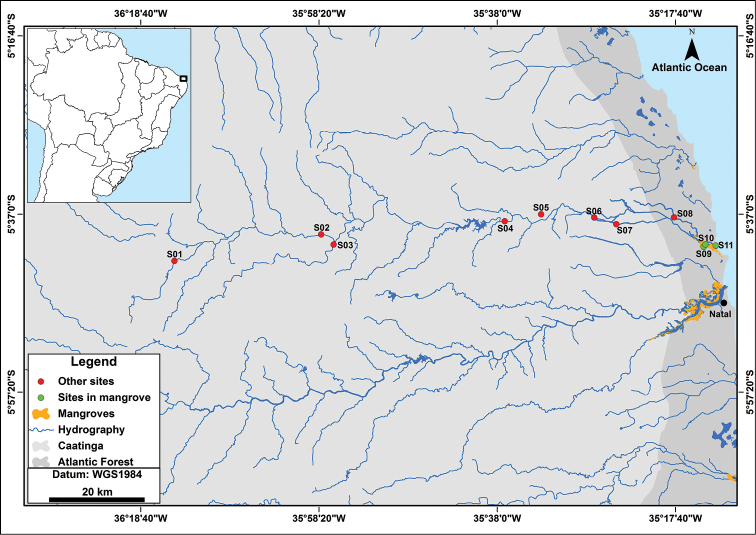
Map of the Ceará-Mirim River basin in Rio Grande do Norte State, northeastern Brazil, showing the sampling sites. Natal, the largest urban center of the state and location of the Potengi River estuary, is indicated by a black dot. Numbers are in accordance to Table 1.

The Ceará-Mirim River basin is approximately 2,770 km², which corresponds roughly to 4.9% of the Rio Grande do Norte State territory (Dias and Fialho 2009) (Figure 1). The main course of the Ceará-Mirim River begins in the municipality of Lajes (05°42'18.4"S, 036°14'49.6"W) and flows for about 120 km in the east direction through the municipalities of Bento Fernandes, Caiçara do Rio do Vento, Ceará-Mirim, Fernando Pedrosa, Jardim de Angicos, João Camara, Pedra Preta, Pedro Avelino, Poço Branco, Riachuelo and Taipu, draining into the ocean at the Extremoz municipality (05°40'33.2"S, 035°13'04.8''W) (Ipea 2016).

Eleven sites (S01-S11) from five municipalities (Lajes, Jardim dos Angicos, Taipu, Ceará-Mirim and Extremoz) were sampled from the headwaters to the estuary of the Ceará-Mirim drainage, including streams, rivers, mangroves and estuary, both at Caatinga (S01-S08) and Atlantic Forest (S09-S11) areas, in order to cover variable microhabitats (Table 1). Two sampling sites (S09-S10) are located at the CTA/UFRN area lower Ceará-Mirim River basin.

**Table 1.** Sampling sites in Ceará-Mirim River basin, Rio Grande do Norte State, northeastern Brazil. CTA/UFRN = "Centro Tecnológico de Aquicultura" (Aquaculture Technological Center) of the Universidade Federal do Rio Grande do Norte.

ID	Municipality	Sampling sites	Biome	Latitude / Longitude
S01	Lajes	River	Caatinga	05°42'18.4"S, 36°14'49.5"W
S02	Jardim de Angicos	River	Caatinga	05°39'17.1"S, 35°58'05.2"W
S03	Jardim de Angicos	Stream	Caatinga	05°40'26.4"S, 35°56'39.6"W
S04	Taipu	River	Caatinga	05°37'46.9"S, 35°37'08.8"W
S05	Taipu	River	Caatinga	05°37'00.0"S, 35°33'00.0"W
S06	Ceará-Mirim	River	Caatinga	05°37'21.6"S, 35°26'56.2"W
S07	Ceará-Mirim	River	Caatinga	05°38'07.4"S, 35°24'24.8"W
S08	Ceará-Mirim	Stream	Caatinga	05°37'20.3"S, 35°17'49.6"W
S09	Extremoz	Mangrove (CTA/UFRN)	Atlantic Forest	05°40'42.6"S, 35°14'27.1"W
S10	Extremoz	Mangrove (CTA/UFRN)	Atlantic Forest	05°40'27.5"S, 35°14'22.9"W
S11	Extremoz	Estuary	Atlantic Forest	05°40'33.5"S, 35°03'05.0"W

### Data Collection

Specimen collections were conducted from June 2011 to September 2016 under permits 30532-1/2011 and 51341-1/2015 provided by ICMBio/SISBIO (Instituto Chico Mendes de Conservação da Biodiversidade/Sistema de Autorização e Informação em Biodiversidade). Fishes were captured using sieves, dip nets, trawl nets, cast nets, and traps. The specimens collected were anesthetized using eugenol, fixed in an aqueous solution of 10% formalin (approximately 8 days) and then preserved in 70% alcohol (Malabarba and Reis 1987). Voucher specimens were deposited at the ichthyologic collection of UFRN. Data from the ichthyologic collections of the California Academy of Sciences which include the Stanford University collections (CAS-SU), and the Universidade Federal da Paraiba (UFPB) were also used in order to qualitatively supplement the species list.

The collected specimens were identified to the lowest taxonomic level possible according to available keys for respective groups (Araújo et al. 2004, Figueiredo and Menezes 1978, 1980, 2000, Jerep and Malabarba 2014, Marceniuk 2005, Menezes and Figueiredo 1980, 1985, Ploeg 1991, Buckup et al. 2007). Some individuals were photographed alive to provide a registry of their live coloration.

Data obtained was compared with the studies of Starks (1913) and Nascimento et al. (2014). The terminology and systematic classification follows Eschmeyer et al. (2016). Habitat details for each species were obtained from *Fishbase* (Froese and Pauly 2016) and *Catalog of Fishes* (Eschmeyer et al. 2016). The conservation status was classified according to the Brazilian lists of endangered species (MMA 2014), and ‘near threatened’ and ‘data deficiency’ species lists (ICMBio 2016). Endemism was defined as species restricted to a single ecoregion according Albert et al. (2011), in this case, the MNCE. Species relevant to artisanal fisheries in coast of Rio Grande do Norte State followed Nóbrega et al. (2015). The classification of introduced species followed Leão et al. (2011).

## Results

**Figure 2.**
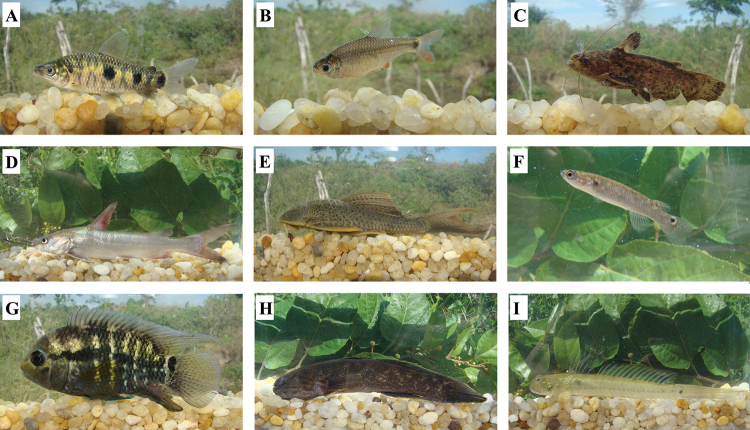
Subset of the ichthyofauna of the Ceará-Mirim River basin, Rio Grande do Norte State, Brazil. **A** *Leporinus piau* **B** *Serrapinnus piaba* **C** *Trachelyopterus galeatus* **D** *Sciades herzbergii* **E** *Hypostomus pusarum* **F** *Kryptolebias hermaphroditus* **G** *Cichlasoma orientale* **H** *Guavina guavina* **I** *Gobionellus oceanicus*.

Specimen collections were conducted along 11 sampling sites (S01-S11) (Table 1, Figure 1) from the upper to the lower Ceará-Mirim River basin, and resulted in the record of 62 fish species, including two non-native (*Oreochromis niloticus* and *Poecilia reticulata*) (Table 2, Figure 2). Based on records for the basin from all the sources consulted (Starks 1913, Nascimento et al. 2014), the species richness is 65, however, *Hoplosternum littorale* probably represents a misidentification (possibly *Megalechis thoracata* Valenciennes) (Table 1).

**Table 2.** Systematic list of fish species of the Ceará-Mirim River basin. Abbreviations: F = freshwater, E = estuarine, M = marine, S = Starks (1913), N = Nascimento et al. (2014), T = this study, DD = deficient data, LC = least concern, NE = not evaluated, NT = near threatened, VU = vulnerable, END = endemic, MIS = misidentification, NNA = non-native, CAS(SU) = Stanford University collections in California Academy of Sciences, UFPB = Universidade Federal da Paraíba, UFRN = Universidade Federal do Rio Grande do Norte. * Importance for artisanal fisheries activities according to Nóbrega et al. (2015), ^+^ recorded in the "Centro Tecnológico de Aquicultura" (Aquaculture Technological Center) of the UFRN.

Order/Family/Species	Habitat	Status	Voucher	S	N	T
**ELOPIFORMES**						
**Elopidae**						
*Elops saurus* Linnaeus, 1766*	M	NE	UFRN 4189			X^+^
**Megalopidae**						
*Megalops atlanticus* Valenciennes, 1847*	M	VU	UFRN 4182			X^+^
**CLUPEIFORMES**						
**Engraulidae**						
*Anchovia clupeoides* (Swainson, 1839)*	M	LC	UFRN 0138			X^+^
*Anchoa januaria* (Steindachner, 1879)	M	LC	UFRN 2661			X^+^
**CHARACIFORMES**						
**Crenuchidae**						
*Characidium bimaculatum* Fowler, 1941	F	LC, END	UFRN 0841			X
**Erythrinidae**						
*Erythrinus erythrinus* (Bloch & Schneider, 1801)	F	LC	UFRN 0082			X
*Hoplias malabaricus* (Bloch, 1794)	F	LC	UFRN 0181	X	X	X
**Anostomidae**						
*Leporinus piau* Fowler, 1941	F	LC	UFRN 0839			X
**Curimatidae**						
*Steindachnerina notonota* (Miranda-Ribeiro, 1937)	F	LC	UFRN 4283	X		X
**Prochilodontidae**						
*Prochilodus brevis* Steindachner, 1875	F	LC	UFPB 9160		X	X
**Serrasalmidae**						
*Metynnis lippincottianus* (Cope, 1870)	F	LC	-		X	
**Characidae**						
Astyanax aff. bimaculatus (Linnaeus, 1758)	F	-	UFRN 0837	X	X	X
Astyanax aff. fasciatus (Cuvier, 1819)	F	-	UFRN 0835		X	X
*Compsura heterura* Eigenmann, 1915	F	LC	UFRN 0846			X
*Cheirodon jaguaribensis* Fowler, 1941	F	DD, END	UFRN 0851			X
*Hemigrammus marginatus* (Ellis, 1911)	F	LC	UFRN 0830		X	X
*Hemigrammus rodwayi* Durbin,1909	F	NE	UFRN 0843			X
*Serrapinnus heterodon* (Eigenmann, 1915)	F	LC	UFRN 0871		X	X
*Serrapinnus piaba* (Lütken, 1875)	F	LC	UFRN 0829	X	X	X
*Serrapinnus potiguar* Jerep & Malabarba, 2014	F	NE, END	UFRN 0870			X
**SILURIFORMES**						
**Auchenipteridae**						
*Trachelyopterus galeatus* (Linnaeus, 1766)	F	LC	-	X	X	X
**Heptapteridae**						
*Rhamdia quelen* (Quoy & Gaimard, 1824)	F	LC	CAS(SU) 22446	X		
**Ariidae**						
*Cathorops arenatus* (Valenciennes, 1840)	E	LC	UFRN 4297			X^+^
*Sciades herzbergii* (Bloch, 1794)*	E	LC	UFRN 4289			X^+^
**Callichthyidae**						
*Hoplosternum littorale* (Hancock, 1828)	F	LC, MIS			X	
**Loricariidae**						
*Hypostomus pusarum* (Starks, 1913)	F	LC, END	UFRN 0842	X		X
**ATHERINIFORMES**						
**Atherinopsidae**						
*Atherinella brasiliensis* (Quoy & Gaimard, 1825)*	M	LC	UFRN 4161			X^+^
**CYPRINODONTIFORMES**						
**Poeciliidae**						
*Poecilia vivipara* Bloch & Schneider,1801	F	LC	UFRN 0073	X	X	X^+^
*Poecilia reticulata* Peters, 1859	F	NNA	UFPB 9162			X
**Cynolebiidae**						
*Kryptolebias hermaphroditus* Costa, 2011	E	NT	UFRN 2475			X^+^
**SYNGNATHIFORMES**						
**Syngnathidae**						
*Hippocampus reidi* Ginsburg, 1933	M	VU	UFRN 2314			X
*Microphis lineatus* (Kaup, 1856)	E	NE	UFRN 4418			X
**SYNBRANCHIFORMES**						
**Synbranchidae**						
Synbranchus aff. marmoratus Bloch, 1795	F	-	UFRN 0186	X		X
**PERCIFORMES**						
**Centropomidae**						
*Centropomus undecimalis* (Bloch, 1792)*	M	LC	UFRN 0132			X^+^
**Serranidae**						
*Mycteroperca bonaci* (Poey,1860)*	M	VU	UFRN 2313			X
*Rypticus* sp.	M	-	UFRN 2310			X
**Lutjanidae**						
*Lutjanus jocu* (Bloch & Schneider, 1801)*	M	NT	UFRN 4409			X
*Lutjanus synagris* (Linnaeus, 1758)*	M	NT	UFRN 4408			X
**Gerreidae**						
*Eucinostomus argenteus* Baird & Girard, 1855*	M	NE	UFRN 0127			X^+^
*Eugerres brasilianus* (Cuvier, 1830)	M	NE	UFRN 0128			X^+^
*Ulaema lefroyi* (Goode, 1874)*	M	NE	UFRN 4135			X^+^
**Mugilidae**						
*Mugil curema* Valenciennes, 1836*	M	DD	UFRN 0129			X^+^
**Cichlidae**						
*Cichlasoma orientale* Kullander, 1983	F	LC	UFRN 0188	X		X
*Crenicichla menezesi* Ploeg, 1991	F	LC	UFRN 0555	X		X
*Oreochromis niloticus* (Linnaeus, 1758)	F	NNA	UFPB 9165			X^+^
**Scaridae**						
*Sparisoma* sp.	M	-	UFRN 4414			X
*Sparisoma radians* (Valenciennes, 1840)	M	LC	UFRN 2312			X
**Eleotridae**						
*Dormitator maculatus* (Bloch, 1792)	E	NT	UFRN 0081			X
*Eleotris pisonis* (Gmelin, 1789)	E	LC	UFRN 4291			X^+^
*Erotelis smaragdus* (Valenciennes, 1837)	E	LC	UFRN 0131			X^+^
*Guavina guavina* (Valenciennes, 1837)	E	LC	UFRN 0088			X^+^
**Gobiidae**						
*Awaous tajasica* (Lichtenstein, 1822)	E	LC	UFRN 0183			X
*Bathygobius soporator* (Valenciennes, 1837)	M	LC	UFRN 4186			X^+^
*Ctenogobius boleosoma* (Jordan & Gilbert, 1882)	E	LC	UFRN 3195			X^+^
*Ctenogobius smaragdus* (Valenciennes, 1837)	E	LC	UFRN 3193			X^+^
*Ctenogobius shufeldti* (Jordan & Eigenmann, 1887)	E	LC	UFRN 3194			X^+^
*Gobionellus oceanicus* (Pallas, 1770)	M	LC	UFRN 0135			X^+^
*Gobioides broussonnetii* Lacepède, 1800.	E	LC	UFRN 3843			X^+^
**Acanthuridae**						
*Acanthurus chirurgus* (Bloch, 1787)*	M	LC	UFRN 4411			X
**Sphyraenidae**						
*Sphyraena* sp.	M	-	UFRN 4417			X
**PLEURONECTIFORMES**						
**Achiridae**						
*Achirus declivis* Chabanaud, 1940	M	LC	UFRN 0868			X^+^
*Achirus lineatus* (Linnaeus, 1758)*	M	LC	UFRN 0191			X^+^
*Trinectes paulistanus* (Miranda Ribeiro, 1915)*	M	LC	UFRN 4298			X^+^
**TETRAODONTIFORMES**						
**Tetraodontidae**						
*Sphoeroides greeleyi* Gilbert, 1900*	E	LC	UFRN 0137			X^+^
*Sphoeroides testudineus* (Linnaeus, 1758)*	E	DD	UFRN 4407			X
**TOTAL**				**11**	**11**	**62**

The 63 native species belong to 54 genera, 32 families and 11 orders. From those species, four (6.3%) are endemic to the MNCE (*Characidium bimaculatum*, *Cheirodon jaguaribensis*, *Hypostomus pusarum* (Figure 2E) and *Serrapinnus potiguar*). Freshwater species represented 38.1% (24 species, excluding two non-native species), estuarine 23.8% (15 species), and marine 38.1% (24 of the total registered species) (Table 2). Among the 39 estuarine and marine species, 17 are important for artisanal fisheries (Nóbrega et al. 2015). Considering only the two sampling sites in the CTA/UFRN (S09-S10) 28 species were caught, including both introduced species, which represents almost half of the species recorded in the basin and 66.6% of the estuarine and marine species (Table 2).

Regarding the conservation status, *Megalops atlanticus* (recorded at S10 location), *Hippocampus reidi* and *Mycteroperca bonaci* (S11) are classified as ‘vulnerable’ (MMA 2014), *Kryptolebias hermaphroditus* (Figure 2F) (S09), *Dormitator maculatus* (S08), *Lutjanus sygnagris* and *L. jocu* (S11) as ‘near threatened’, while *Cheirodon jaguaribensis* (S05, S07 and S08), *Mugil curema* (S09 and 10) and *Sphoeroides testudineus* (S11) are listed as ‘data deficient’ (ICMBio 2016). Among those species, only *C. jaguaribensis* is a freshwater species. The remaining species are currently classified as ‘least concern’ or were not evaluated (Table 2).

## Discussion

This study reports 63 native and two introduced species in the Ceará-Mirim River basin, adding 48 species to the previous lists provided by Starks (1913) and Nascimento et al. (2014). Both studies mentioned only 11 species, all freshwater species, even though only five were common to both lists (Astyanax aff. bimaculatus, *Hoplias malabaricus*, *Poecilia vivipara*, *Serrapinnus piaba*, and *Trachelyopterus galeatus*). We recorded 26 freshwater species, nine of which are new records, including two non-native species (*Oreochromis niloticus* and *Poecilia reticulata*). The discrepancy between our species count data and those from previous studies may be due to the use of selective fishing gear in the earlier studies, or the small number of microhabitats explored. Starks (1913) and Nascimento et al. (2014) did not identify the same taxa in their work. Starks (1913) collected specimens of *Cichlasoma orientale* (Figure 2G), *Crenicichla menezesi*, *Hypostomus pusarum*, *Rhamdia quelen*, *Steindachnerina notonota* and Synbranchus aff. marmoratus. In turn, Nascimento et al. (2014) listed Astyanax aff. fasciatus, *Hemigrammus marginatus*, *Hoplosternum littorale*, *Metynnis lippincottianus*, *Prochilodus brevis* and *Serrapinnus heterodon*, all not mentioned by Starks (1913), even when updating the taxonomic identification (Eschmeyer et al. 2016).

Among the species listed by Starks (1913), *Rhamdia quelen* was not found in our field surveys, and *Hoplosternum littorale* and *Metynnis lippincottianus*, present in both lists (Starks 1913, Nascimento et al. 2014) were also not collected. With the exception of *H. littorale*, that is not known in the MNCE, and could actually represent a misdentification (the species might actually be *Megalechis thoracata*, the callichthyid known to occur in MNCE), all species not listed in our collections were recently recorded in a nearby coastal basin (Paiva et al. 2014) and may occur in the Ceará-Mirim drainage.

Although Starks (1913) did not mention any marine or estuarine fish among the Ceará-Mirim River basin material, he listed 79 marine and ten estuarine species at the municipality of Natal, some probably obtained in the Potengi River estuary, but also in intertidal rock pools and local markets. Among those, seven marine (*Achirus lineatus*, *Eugerres brasilianus*, *Erotelis smaragdus*, *Lutjanus jocu*, *L. synagris*, *Mugil curema* and *Ulaema lefroyi*), and two estuarine species (*Ctenogobius boleosoma* and *Spheroides testudineus*) were also recorded at Ceará-Mirim River basin. Due to the proximity of the above-mentioned estuaries (about 9 km), the presence of the other species in Ceará-Mirim River cannot be ruled out. In this study, the collections in the estuary were carried out in flooded areas of the mangrove forest, an area that is usually poorly sampled in ichthyological surveys, while in other studies, especially Starks (1913), they were probably performed on the main channel of the estuary resulting in the collection of larger species. In addition, the species collected in the present study are small, as well as juveniles of other marine fish species, emphasizing the role of the mangroves in fish recruitment and reproduction (Osório et al. 2011).

Sales et al. (2016) stressed the importance of hypersaline estuaries at the north coast of Rio Grande do Norte State as nurseries for reef fish, and listed 45 estuarine and 33 marine, respectively. Although putatively widely distributed along Brazilian estuaries, the following species found in our surveys were not registered by Sales et al. (2016): estuarine, *Awaous tajasica*, *Cathorops arenatus*, *Ctenogobius shufeldti*, *Eleotris pisonis*, *Erotelis smaragdus*, *Gobioides broussonnetii*, *Guavina guavina*, *Kryptolebias hermaphroditus*, *Microphis lineatus* and *Sciades herzbergii*; marine, *Achirus declivis*, *Anchovia clupeoides*, *Anchoviella lepidentostole*, *Atherinella brasiliensis*, *Elops saurus*, *Gobionellus oceanicus*, *Megalops atlanticus*, *Rypticus* sp., *Sparisoma* sp., *Sphyraena* sp, *Sparisoma radians* and *Trinectes paulistanus*. Among the marine species recorded in the Ceará-Mirim River basin, *Megalops atlanticus* and *Mycteroperca bonaci* are classified as ‘vulnerable’. However they are also considered important for artisanal fisheries in Rio Grande do Norte State (Nóbrega et al. 2015). In both cases only juvenile individuals were registered, corroborating Araújo et al. (2002) in the importance of coastal basins, even the small ones, for the maintenance of fish stocks and life cycles of several marine species that use estuaries as nurseries.

According to Blaber and Barletta (2016), it was only over the last 40 years that more detailed studies involving estuarine fish started to be conducted. This data deficiency may be linked to logistical difficulties (e.g. use of inappropriate fishing gear, access and dislocation in the muddy substrate and through its complex structure), taxonomic difficulties, financing sources and research infrastructure. The Ceará-Mirim River estuary has a small area when compared to the whole extent of the basin (0.12%, Lira et al. 2015), but it harbors 38 (60.3%) of the 63 native fish species of the drainage. Among those species, only *Poecilia vivipara* is considered as freshwater, although it shows tolerance to saline environments (Gomes 2008).

At the Ceará-Mirim River basin, environmental impacts caused by inadequate use of soil, irregular human occupation of sand dunes and mangrove areas, deficiencies in wastewater treatment systems, as well as marginal deforestation have been reported by Soares et al. (2010). Such impacts may negatively affect the most sensitive species such as the endangered seahorse *Hippocampus reidi* (MMA 2014) and the ‘piaba’ *Serrapinnus potiguar*, a species recently described for the Ceará-Mirim River basin (Jerep and Malabarba 2014). Blaber and Barlleta (2016) mentioned the pollution caused by industrialization, intensive agriculture and climate change as major anthropogenic effects affecting estuaries. Due to climatic changes some introduced freshwater species with high tolerance to salinity, such as the tilapia *Oreochromis niloticus* recorded in the lower reaches of the Ceará-Mirim River basin, might increase their chances of dispersion and establishment in neighboring basins by dislocation among estuaries (Guttiere et al. 2014).

This survey of the Ceará-Mirim River basin’s ichthyofauna can be an useful tool contributing to further academic activities and environmental education, including making local inhabitants aware of the need to preserve the diversity of fish in the coastal basins of Brazil, highly modified by the irregular occupation and unregulated tourism in northeastern Brazil. The CTA/UFRN, although not a conservation unit, may represent an important area for the recovering of the mangrove vegetation and maintenance of estuarine and marine fish species, some of them endangered and commercially exploited.
